# Unveiling the Veil: Exploring Diabetic Retinopathy Awareness and Behaviors of the General Population of Hafr Al Batin, Saudi Arabia

**DOI:** 10.7759/cureus.48629

**Published:** 2023-11-10

**Authors:** Farhan K Alswailmi

**Affiliations:** 1 Department of Pharmacy Practice, College of Pharmacy, University of Hafr Al Batin, Hafr Al Batin, SAU

**Keywords:** saudi arabia, practice, knowledge, attitude, diabetic retinopathy

## Abstract

Background: Diabetic retinopathy (DR) is a potentially blinding complication of diabetes mellitus (DM). The current study set out to explore the awareness level and attitude of the general population of Hafr Al Batin, Saudi Arabia, where DM prevalence is high.

Methodology: Data was collected through community-based, self-administered questionnaires in the general population of Hafr Al Batin, Saudi Arabia. The study included 406 participants, and the analysis revealed varying levels of knowledge, attitude, and practices related to DR. The study also explored the associations between sociodemographic factors and knowledge, attitude, and practices related to DR. The analysis showed that the participants had medium (67.4%) knowledge levels, while 32.5% had low knowledge.

Results: The participants comprised 55.7% males and 44.3% females, with varying education levels and economic statuses. The majority were diagnosed with diabetes (94.3%). Attitude scores revealed that 30.8% had a favorable attitude, and 69.2% had medium attitude levels. Regarding practices, 71.2% of the respondents had low practice levels. The association between sociodemographic factors and knowledge, attitude, and practice were also explored.

Conclusion: The current study concluded a medium level of knowledge (67.4%), and attitude toward DR is also low (69.2%). More than half of the respondents followed a poor level of practice (71.2%). The results of the current investigation demonstrated that the general population does not have sufficient knowledge, attitude, and practices regarding DR in Saudi Arabia. By promoting greater knowledge and understanding of DR, the burden of visual impairment brought on by this complication can be lessened by early detection and efficient care.

## Introduction

According to the International Diabetes Federation (IDF), the prevalence of diabetes mellitus (DM) among adults in the Kingdom of Saudi Arabia (KSA) is 18.3%, and there are an estimated 4.3 million cases of DM globally [1]. Diabetic retinopathy (DR) is one of the most prevalent consequences of DM and one of the many complications that can result from DM [2,3]. In today’s population with DM, DR is thought to be the most prevalent and dangerous microvascular consequence that causes blindness and visual impairment [4,5]. The reason for blindness includes diabetic maculopathy, vitreous hemorrhage, retinal detachment, and neovascular glaucoma [6,7].

DM patients around the world have a prevalence of DR ranging from 18.9% to 40.3% [8,9]. After more than 15 years with the condition, between 78% and 97% of type 1 diabetes patients and between 60% and 80% of type 2 diabetic patients will experience some degree of DR [10]. According to research, DR is the third most common culprit of sight loss in Saudi Arabia [11]. In Saudi Arabia, a large proportion of diabetic patients experience DR. The reported prevalence of DR in the Al-Ahsa region is 33% [12]. Almost the same proportion (36%) is reported in the Medina region [13].

Despite the high rate of DR-related blindness, delaying the start of the disease and slowing its course require early detection and the implementation of efficient screening programs and measures to manage DR-related risk factors. Diabetes patients must control their hypertension, blood glucose, and lipid levels to halt the advancement of ocular problems [14]. Additionally, regular eye examinations and timely interventions are crucial. If patients are managed promptly and effectively, current treatment options have been shown to prevent up to 98% of vision loss and blindness caused by severe retinopathy [15]. To implement an effective health awareness strategy for DR, it is important to work on knowledge, attitude, and practices of diabetic patients toward ocular problems and care.

The existing literature lacks sufficient information on the awareness of DR among individuals with diabetes. To the best of our knowledge, limited studies have been conducted with regard to the behavioral awareness of DR in Saudi Arabia. Therefore, this study was undertaken to assess the knowledge, attitude, and practices related to DR among the general population in Saudi Arabia.

## Materials and methods

Study design

A cross-sectional study was conducted in the general population of Hafr Al Batin, Saudi Arabia. Data was collected through community-based, self-administered questionnaires from January to September 2023. The study followed the Declaration of Helsinki, and ethics approval was obtained from the ethical committee of the University of Hafr Al Batin, Hafr Al Batin, Saudi Arabia (0015-1443-S).

Inclusion and exclusion criteria

People who were 18 years and above were included in the study. People who were mentally and physically impaired were excluded from the study.

Sample size calculation

The sample size for our study was determined using Raosoft software (Raosoft, Inc., Seattle, WA), specifically using the single proportion sample size formula. We aimed for a precision of 5% and a confidence interval (CI) of 95%. The assumed percentage of diabetic patients with significant knowledge was >60%.

Data collection tool

Patients were asked to complete the self-administered questionnaires. The survey was spread through social media, WhatsApp and Facebook. The questionnaire was translated into Arabic. Ophthalmology specialists with at least 10 years of experience assessed the questionnaire to determine its content validity. A pilot study was conducted with a representative population who were not part of the main study. The questionnaire included demographic information such as age, gender, education level, economic status, and duration of diabetes. Systemic comorbidities such as cardiovascular diseases, dyslipidemia, and hypertension were also taken into account. It also included questions to assess the participant’s knowledge, attitude, and practices related to retinopathy, their choice of healthcare professional, and treatment options for diabetic retinopathy. For the knowledge, attitude, and practice sections, a five-point Likert scale with responses ranging from strongly agree to strongly disagree was recorded. One point was given for strongly disagree and five for strongly agree. Low (60% of the total score), medium (61%-80% of the total score), and high (>80% of the total score) were the categories used to classify the total scores for each section.

Statistical analysis

Statistical analysis was performed using Statistical Package for the Social Sciences (SPSS) version 28 (IBM SPSS Statistics, Armonk, NY), utilizing descriptive and inferential statistics [16]. The association between knowledge, attitude, and practice and DR was measured using logistic regression (univariate and multivariate).

## Results

Socioeconomic characteristics

In the sample, there were 226 (55.7%) males and 180 (44.3%) females. The age range of the majority of respondents was 18-28 (225, 55.4%), followed by 29-39 (86, 21.2%), 40-49 (82, 20.2%), and 50 and older (13, 3.2%). Eleven (2.7%) of the respondents had finished high school, and 90 (22.2%) had graduated from high school. A total of 222 (54.7%) respondents had earned a bachelor’s degree, 68 (16.7%) had received a diploma, and 15 (3.7%) had earned a master’s degree or PhD. In addition, 383 (94.3%) respondents had been given a diabetes diagnosis, while 23 (5.7%) did not (Table 1).

**Table 1:** Sociodemographic characteristics of the general population (N=406) BMI: body mass index

Variables	Frequency	%
Gender		
Male	226	55.7
Female	180	44.3
Age (years)		
18-28	225	55.4
29-39	86	21.2
40-49	82	20.2
50 and more	13	3.2
BMI (kg/m^2^)		
<18.5	19	4.7
18.5-30	50	12.3
>30	100	24.6
I don’t know	237	58.4
Education		
Secondary school	11	2.7
High school	90	22.2
Bachelor’s degree	222	54.7
Diploma	68	16.7
Master’s degree/PhD	15	3.7
Occupation		
Student	142	35
Employed	176	43.3
Unemployed	88	21.7
Marital status		
Single	192	47.3
Married	214	52.7

Type of respondents 

**Figure 1:**
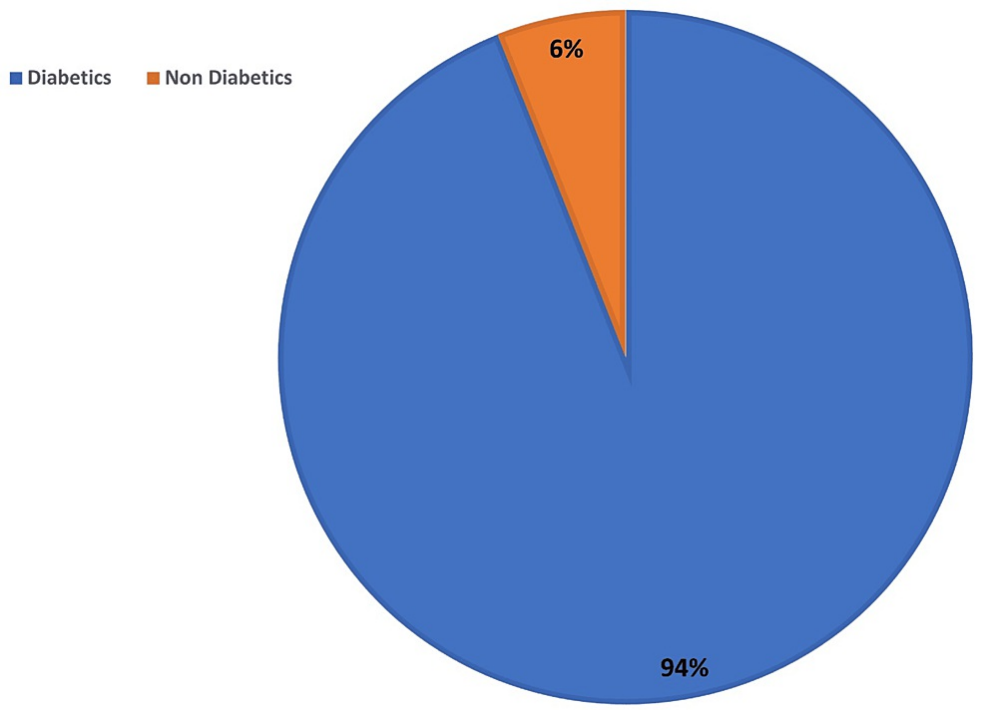
Percentage of respondents in the study

In our study, the majority of the population was diabetic. Around 383 (94%) respondents were diabetic, and 23 (6%) were non-diabetic (Figure 1).

Frequency of eye checkups

As mentioned in Table 2, most of the respondents (52.9%) did not go for an eye evaluation, 30.5% went once in six months, and only 0.98% went to the eye doctor monthly.

**Table 2:** Frequency of eye checkups

Eye examination	Number	%
Monthly	4	0.98
Once in six months	63	15.5
Yearly	124	30.5
Never	215	52.9

Source of awareness regarding DR

**Figure 2:**
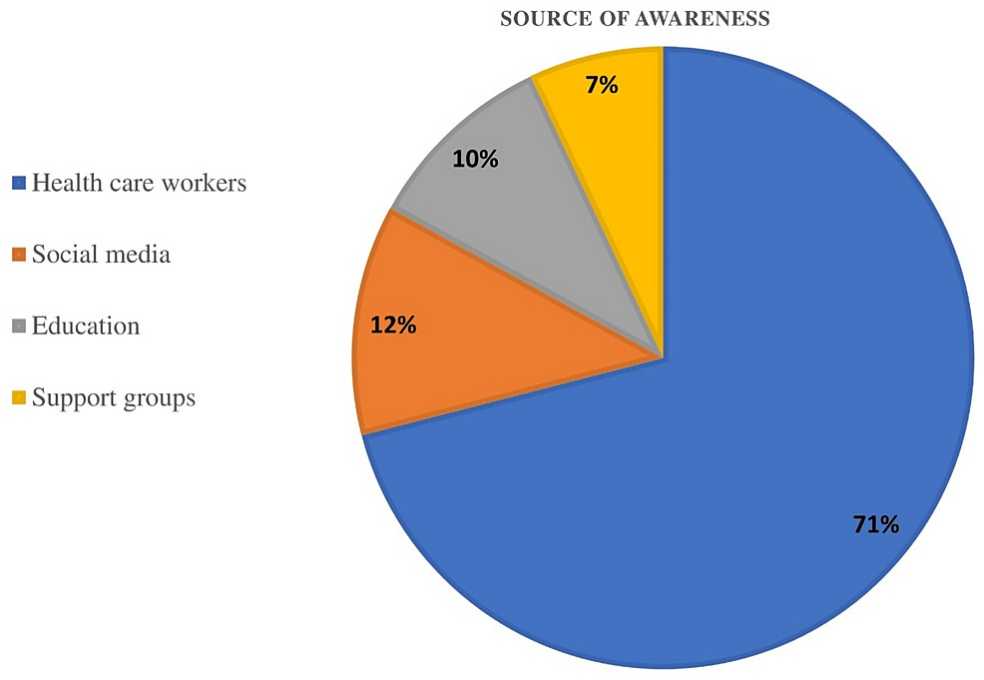
Source of information regarding DR DR: diabetic retinopathy

Figure 2 shows the data regarding the awareness of DR. The majority of the respondents who have knowledge about DR have been made aware through healthcare workers (71%). Social media also played an important role (12%). Of the respondents, 10% were made aware by education, and 7% have known DR through support groups.

The findings shown in Table 3 suggest that a significant portion of the surveyed population has a medium level of knowledge (67.4%). A substantial proportion of respondents have a medium level of attitude (69.2%) and a low level of practice. Of the respondents, 71.2% do not follow good practices for DR.

**Table 3:** Knowledge, attitude, and practice regarding diabetic retinopathy among the general population (N=406)

Variables	Number	%
Knowledge		
High	274	67.4
Medium/low	132	32.5
Attitude		
High	125	30.8
Medium/low	281	69.2
Practice		
High	117	28.8
Medium/low	289	71.2

Association between knowledge, attitude, and practice and diabetic retinopathy

The average knowledge score for males was 45.50±6.20, with a significant p-value of 0.02. The mean attitude score was 31.43±5.86, with a highly significant p-value of 0.001. The mean practice score was 41.32±4.98, with a significant p-value of 0.007. The mean knowledge score of people who have attended secondary school was 46.15±4.90, with a significant p-value of 0.01. The mean attitude score was 31.43±6.80, with a highly significant p-value of 0.007. The mean practice score was 42.87±4.23, with a highly significant p-value of 0.001. The results are shown in Table 4.

**Table 4:** Scores of knowledge, attitude, and practice regarding DR and the sociodemographic characteristics of the study participants DR: diabetic retinopathy, SD: standard deviation, BMI: body mass index

Variables	Knowledge		Attitude		Practice	p-value
	Mean±SD	p-value	Mean±SD	p-value	Mean±SD	
Gender						
Male	45.50±6.20	0.02	31.43±5.86	0.001	41.32±4.98	0.007
Female	45.30±5.35		32.89±5.29		41.90±4.90	
Age (years)						
18-28	45.25±4.58	0.21	32.23±5.29	0.12	42.21±4.89	0.98
29-39	45.22±4.81		34.21±5.31		43.43±4.65	
40-49	43.46±4.14		32.67±5.21		42.36±4.23	
50 and more	43.85±4.57		35.12±5.45		41.67±4.21	
BMI (kg/m^2^)						
<18.5	41.55±5.98	0.32	31.90±5.21	0.42	41.32±4.43	0.21
18.5-30	42.90±5.90		31.32±5.84		41.65±4.16	
>30	41.46±5.10		32.12±5.09		43.75±4.76	
I don’t know	45.45±5.51		34.45±5.12		41.43±4.87	
Education						
Secondary school	46.15±4.90	0.01	31.43±6.80	0.007	42.87±4.23	0.001
High school	42.32±4.85		32.21±5.12		41.12±4.75	
Bachelor’s degree	43.11±5.34		32.20±5.90		41.31±4.21	
Diploma	41.40±5.98		33.12±5.11		42.12±4.45	
Master’s degree/PhD	40.21±5.57		31.80±5.11		41.78±4.54	
Occupation						
Student	41.78±5.96	0.005	31.20±6.81	0.05	42.65±4.97	0.001
Employed	44.54±5.32		31.20±6.81		41.12±4.91	
Unemployed	44.12±5.78		32.20±6.81		41.87±4.95	
Marital status						
Single	41.09±5.21	0.41	32.20±6.81	0.92	41.23±4.21	0.67
Married	44.34±4.09		33.20±6.81		41.54±4.17	
Respondents						
Diabetics	41.85±5.87	0.001	33.20±6.81	0.001	42.32±4.65	0.001
Non-diabetics	40.90±5.21		33.20±6.81		42.45±4.43	

Association between knowledge, attitude, and practice and diabetic retinopathy by logistic regression

In the univariate analysis, higher knowledge was associated with an odds ratio (OR) of 1.2 (95% confidence interval (CI): 1.1-1.8). In the multivariate analysis, after adjusting for other variables, higher knowledge was associated with a decreased adjusted odds ratio (AOR) of 0.7 (95% CI: 0.1-0.8), with a statistically significant p-value of 0.03. In the univariate analysis, a more positive attitude was associated with an OR of 1.5 (95% CI: 1.3-1.9), with a statistically significant p-value of 0.01. In the multivariate analysis, after adjusting for other variables, a more positive attitude was associated with a decreased AOR of 0.6 (95% CI: 0.1-0.9), with a statistically significant p-value of 0.01 as displayed in Table 5.

**Table 5:** Association between knowledge, attitude, and practice toward diabetic retinopathy by logistic regression OR: odds ratio, AOR: adjusted odds ratio, CI: confidence interval

Variables	Univariate analysis		Multivariate analysis	
	OR (95% CI)	p-value	AOR (95% CI)	p-value
Knowledge	1.2 (1.1-1.8)	0.05	0.7 (0.1-0.8)	0.03
Attitude	1.5 (1.3-1.9)	0.01	0.6 (0.1-0.9)	0.01

## Discussion

The current study set out to explore the awareness level and attitude of the general population in Hafr Al Batin, Saudi Arabia. To the best of our knowledge, no study has reported on the awareness level and attitude of the general population in Hafr Al Batin, Saudi Arabia, toward DR. Our study concluded a medium level of knowledge (67.4%) and attitude (69.2%). More than half of the respondents followed a poor level of practice (71.2%). DR is one of the most common causes of visual impairment worldwide; however, a big section of the public is largely unaware of it. The results of the current investigation demonstrated that the general population in Saudi Arabia does not have sufficient knowledge, attitude, and practices regarding DR.

The knowledge rate of the participants was 67.4%. This rate is slightly higher than the knowledge rates in India (50%) [17] and the United States (52%) [18]. However, it lagged behind other nations such as Australia (96%) [19] and Japan (98%) [20]. This gap is a result of the differing healthcare systems and literacy rates between these two countries. The most prevalent public health issue in the 21st century is DM [21]. Despite having a significant negative impact on resources and human well-being, chronic diseases such as DM continue to be disregarded by many countries. The number of people with DM is rising as a result of population growth, urbanization, the spread of obesity, and physical inertia [22]. Due to ignorance of their condition, people with DM may have several problems such as hypertension and DR. There is growing evidence that patient education is the most effective strategy for easing the complications brought on by DM [22]. The major consequence of DM is DR. Around 1.8 million people globally are known to go blind as a result of DR, if not treated properly [23]. Periodic eye assessments for complications and timely management of DM patients can delay or reduce the complexity of DR by up to 50% [24]. The prevalence of DR can be decreased by managing blood sugar levels and scheduling routine eye examinations [25].

Building an effective program to combat any disease in the community must start with educating the people, and this is especially true for the rising issue of DR. To increase public awareness of diabetes and visual impairment in diabetic patients, it is essential to understand the gaps in knowledge, attitudes, and behaviors in this area.

In the current study, we discovered that just 30.8% of the participants had a favorable attitude toward DR. In contrast, Hussain et al. [26] discovered that 53.8% of DM patients in their study had a favorable attitude toward DR, and 158 (54.9%) patients had good diabetic practice patterns. In contrast, it was revealed that of the subjects in the investigations by Hussain et al. [26] and Rani et al. [27], 57.6% and 48.45% of the patients, respectively, had good practice routines.

The participants of the current study showed low practice (28.8%) toward DR, which pointed out that the practice percentage of DR fell below the desirable range. This can be explained by the population’s poor attitude and knowledge levels. Although DM patients may be well-informed about the consequences of DR, this does not imply that they will be well-practiced. The lack of good knowledge and the presence of negative attitudes within a community suffering from DR can be attributed to several social causes, such as limited healthcare access, cultural stigma, fear, and anxiety. These factors often contribute to delayed diagnosis, poor management, and increased risk of complications associated with DR. In the study by Funatsu et al. [28], more than 98% of patients knew they had DR, but only 69.5% of them were willing to consult an ophthalmologist for routine eye examinations. Moreover, Alzahrani et al. [29] reported a similar finding, showing that only 65% of their sample received frequent eye examinations even though 82.6% of them were aware of DR.

We looked at the connections between factors such as gender, age, body mass index (BMI), education, occupation, and marital status and the following three main aspects: knowledge, attitude, and practice. Concerning knowledge, with a significant p-value of 0.02, males outperform females in terms of mean knowledge scores (45.50 versus 45.30). The mean attitude score for males is similarly considerably higher than for females (31.43 versus 32.89), with a p-value of 0.001. With a significant p-value of 0.007, males outperform females in terms of mean practice score (41.32 versus 41.90). In line with this, Mahesh et al. [30] discovered a statistically significant correlation between knowledge of retinopathy and retinopathy practice. In contrast to our study, age and gender were shown to be strongly associated with previous findings regarding understanding of DR [31,32]. Age, however, had no statistically significant relationship with knowledge of DR in the current investigation.

With a significant p-value of 0.01 and the highest mean knowledge score, respondents with secondary education are the most knowledgeable. In terms of attitude, individuals who have completed secondary school had the highest mean scores, with a significant p-value of 0.007.

Individuals who have completed secondary school have the highest mean practice scores, with a significant p-value of 0.001. Students have a significantly higher mean knowledge score compared to employed and unemployed individuals (p=0.005). Students also have a significantly higher mean attitude score compared to employed individuals (p=0.05). Students have a significantly higher mean practice score compared to both employed and unemployed individuals (p=0.001). These results are in line with the study conducted in Ghana and India [30,31].

Diabetics have significantly higher scores in knowledge, attitude, and practice compared to non-diabetics (p=0.001). The findings imply that differences in knowledge, attitude, and practice linked to the topic under study are related to factors such as gender, education, and occupation. In addition, compared to respondents without diabetes, respondents with diabetes typically score higher in each of the three categories. Age, BMI, and marital status do not appear to be major variables that influence these features in this study.

Patients from all demographic groups participated in this study, which was conducted at several different centers. It was constrained by a questionnaire based on the glycemic control level.

Limitations

This is a single-center study, and thus, this study may have limited access to comprehensive data. The findings may not be applicable to other centers or diverse patient populations. Retrospective studies rely on data collected from medical records, which may introduce selection bias. The study population may not be representative of the entire patient population, as certain patients or cases may be excluded or underrepresented.

## Conclusions

Our study concluded a medium level of knowledge (67.4%) and attitude (69.2%). More than half of the respondents followed a poor level of practice (71.2%). DR is one of the most common causes of visual impairment worldwide; however, a big section of the public is largely unaware of it. The results of the current investigation demonstrated that the general population in Saudi Arabia does not have sufficient knowledge, attitude, and practices regarding DR. This baseline information on knowledge, attitude, and practice about eye care among diabetics should be considered and given special attention when conducting health promotion initiatives. Special outreach and educational efforts are needed to raise awareness of diabetics, in particular those who are at an increased risk of poor knowledge, attitude, and practices. Also, there is a greater need to focus on non-diabetic respondents.
